# Peripheral Lymphatic Shunts for Protein-Losing Enteropathy in a Child with THSD1 Mutation: A Case Report

**DOI:** 10.1055/a-2777-5624

**Published:** 2026-03-03

**Authors:** Hatan Mortada, Feras Alshomer, Hyungjoo Noh, Changsik John Park, Joon Pio Hong

**Affiliations:** 1 Division of Plastic Surgery, Department of Surgery, King Saud University Medical City, King Saud University, Riyadh, Saudi Arabia; 2 Department of Plastic Surgery and Burn Unit, King Saud Medical City, Riyadh, Saudi Arabia; 3 Division of Plastic Surgery, Department of Surgery, King Saud bin Abdulaziz University for Health and Sciences, Riyadh, Saudi Arabia; 4 Beauty for Life Center in Yas Clinic-Abu Dhabi Stem Cell Center (ADSCC), Abu Dhabi, United Arab Emirates; 5 Department of Plastic and Reconstructive Surgery, University of Ulsan College of Medicine, Asan Medical Center, Seoul, South Korea

**Keywords:** protein-losing enteropathy, THSD1 mutation, lymphatic dysfunction, lymph–venous bypass, lymph node to vein anastomosis, hypoalbuminemia, pediatric lymphedema, lymphatic surgery, lymphoscintigraphy

## Abstract

Protein-losing enteropathy (PLE) is a rare disorder characterized by abnormal protein loss through the gastrointestinal tract, often leading to hypoalbuminemia and malnutrition. This case report describes the successful surgical management of chronic lower limb lymphedema and PLE in a 7-year-old male with a thrombospondin type 1 domain-containing 1 (THSD1) mutation, unresponsive to conventional therapies. Despite aggressive nutritional support, including albumin infusions, the patient experienced persistent hypoalbuminemia and ongoing protein loss. Imaging revealed significant lymphatic dysfunction, prompting lymphovenous bypass and lymph node–vein anastomosis (LNVA). Postoperatively, serum albumin levels improved from 1.5 to 3.2 g/dL, limb circumference decreased, and alpha-1 antitrypsin levels normalized. However, a 3-year follow-up revealed a relapse of hypoalbuminemia following an upper respiratory infection, underscoring the need for additional interventions in growing pediatric patients. This case strengthens the potential of lymphatic surgery in addressing PLE-related lymphatic dysfunction when medical treatments fail and emphasizes the need for further research to confirm the efficacy of this approach.

## Introduction

Protein-losing enteropathy (PLE) is a rare but severe condition characterized by abnormal protein loss through the gastrointestinal tract, leading to hypoalbuminemia and systemic complications such as edema and malnutrition. 1 While PLE can arise from various causes, lymphatic dysfunction is increasingly recognized as a significant underlying factor, especially in congenital or genetic lymphatic malformations. 2 Primary intestinal lymphangiectasia, for instance, involves the abnormal dilation and rupture of enteric lymphatic vessels, causing protein loss and lymphopenia. 3 Lymphangiectasia, the pathological enlargement of lymphatic vessels due to lymphatic failure or obstruction, results in leakage into surrounding tissues or the gastrointestinal tract. This lymphatic dysfunction can cause conditions like PLE, marked by protein loss, hypoalbuminemia, and edema. Lymphangiectasia plays a critical role in several lymphatic disorders, influencing both diagnosis and treatment strategies. 1 4 The condition illustrates the complexities of diagnosing and managing PLE, as lymphatic anomalies are often overlooked or misdiagnosed in early stages. 5 6

Lymphatic surgery, particularly techniques such as lymphovenous bypass and LNVA, has emerged as a promising therapeutic option for addressing lymphatic dysfunction at various stages of lower extremity lymphedema. 7 Previous reports have shed light on the potential applications of surgical interventions like LNVA and lymphovenous bypass in managing PLE. For example, Jamal reported successful management of PLE associated with peripheral lymphedema using a combination of lymph node–vein shunt and soft tissue debulking surgery for lower extremity and scrotal edema. 8 Similarly, Miyakawa et al described a case of intestinal lymphangiectasia with refractory gastrointestinal bleeding, where intra-abdominal lymphovenous anastomosis (LVA) and venous ligation led to significant improvement. 9

To the best of our knowledge, comprehensive data on the incidence of thrombospondin type 1 domain-containing 1 ( *THSD1* ) gene mutations, particularly in relation to primary lymphedema and PLE, are lacking in the current literature. This case report details the successful application of lymphovenous bypass and LNVA in a 7-year-old male with a THSD1 mutation, who presented with chronic PLE and lower limb lymphedema, both unresponsive to conventional medical treatment.

## Case

### Case Presentation

A 7-year-old male, born at 37 weeks of gestation with a known THSD1 mutation, presented with chronic PLE and lower limb lymphedema. His neonatal history includes neonatal intensive care unit (NICU) admission for transient tachypnea, requiring biphasic CPAP support, as well as bilateral inguinal hernia repair and hydrocelectomy. He also has asthma, managed with a fluticasone inhaler and Singulair. The patient had a long-standing history of gastrointestinal symptoms, including diarrhea, abdominal distention, and peripheral edema. Laboratory evaluations consistently revealed severe hypoalbuminemia, with serum albumin levels ranging from 1.5 to 2.0 g/dL (normal: 3.5–5.5 g/dL), leading to significant protein loss and malnutrition. Despite ongoing medical management, including weekly albumin infusions and a high-protein diet, the patient's symptoms persisted and progressively worsened. Prior to surgery, the patient received complete decongestive therapy, which failed to provide lasting clinical improvement. Written informed consent was obtained from the patient's legal guardian for publication of the clinical data and images included in this report.

### Genetic, Clinical Background, and Diagnostic Workup

**Fig. 1:**
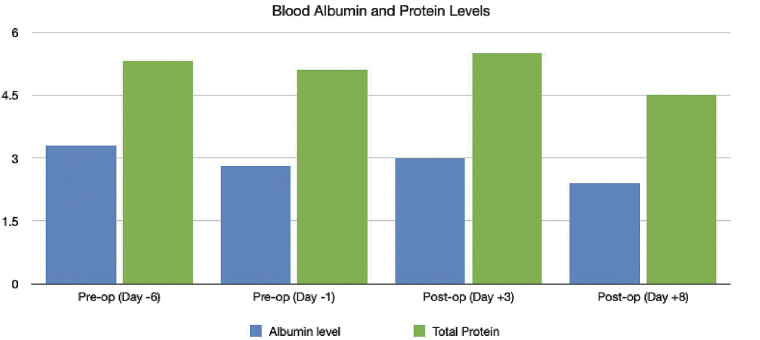
Blood albumin and total protein levels measured at four time points relative to surgery: Preoperative day −6 and day −1, and postoperative day +3 and day +8.

**Fig. 2:**
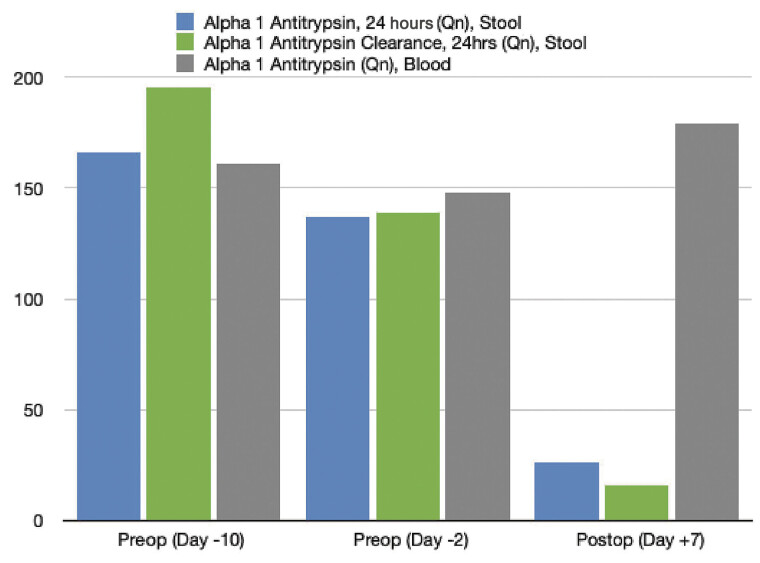
Changes in alpha-1 antitrypsin levels and clearance pre- and postoperatively.

**Fig. 3:**
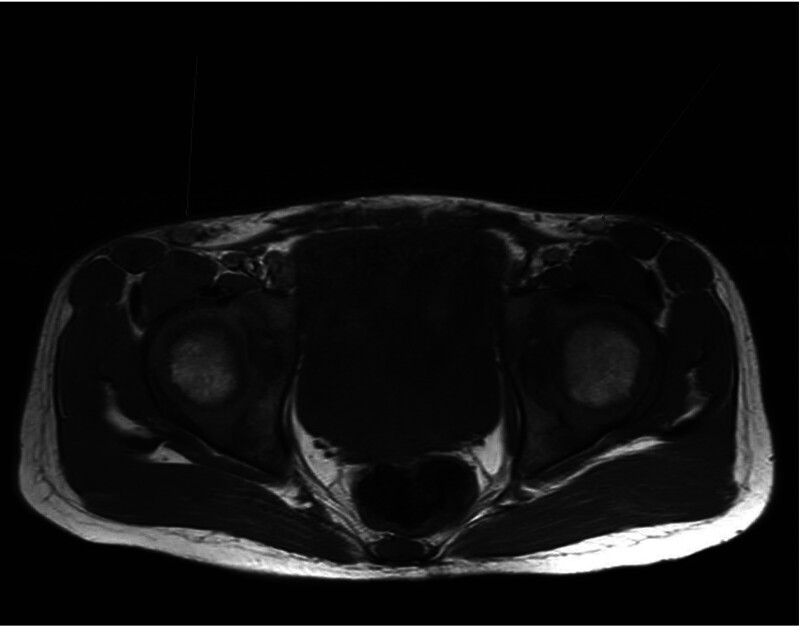
Axial T1-weighted MRI of the pelvis without contrast showing multiple benign-appearing lymph nodes (arrows) in the bilateral inguinal regions. No evidence of pathological lymphadenopathy or osseous lesions was noted. The imaging was performed as part of the preoperative assessment for lower limb lymphedema and protein-losing enteropathy.

Despite aggressive nutritional therapies, including albumin infusions and diuretics to manage edema, his serum albumin levels remained persistently low, between 1.5 and 2.0 g/dL. Additional laboratory tests showed low total protein levels (5.1–5.5 g/dL), and alpha-1 antitrypsin clearance was markedly elevated at 138.7 to 195.6 mg/dL (normal: <27 mg/dL; Figs. 1 and 2 ). Immunoglobulin levels were closely monitored, with IgG fluctuating between 593.5 and 385.9 mg/dL, and IgM ranging from 49.8 to 35.2 mg/dL. Imaging studies, including lymphoscintigraphy, MRI, and MR lymphangiography, revealed abnormal lymphatic flow, with lymphatic leakage in the abdomen, bilateral foot leakage, and narrowing at the thoracoabdominal junction of the thoracic duct ( Figs. 3 4 5 ). Medical and rehabilitation therapies failed to stop the progression of the disease. Given the patient's worsening PLE, lower limb lymphedema, and hypoalbuminemia (as low as 1.5 g/dL), surgical intervention was deemed necessary ( Fig. 6 ). Preoperative management included complex decongestive therapy and compression, which did not lead to sustained clinical improvement. Postoperatively, CDT was withheld to preserve the integrity of the lymphatic anastomoses.

**Fig. 4:**
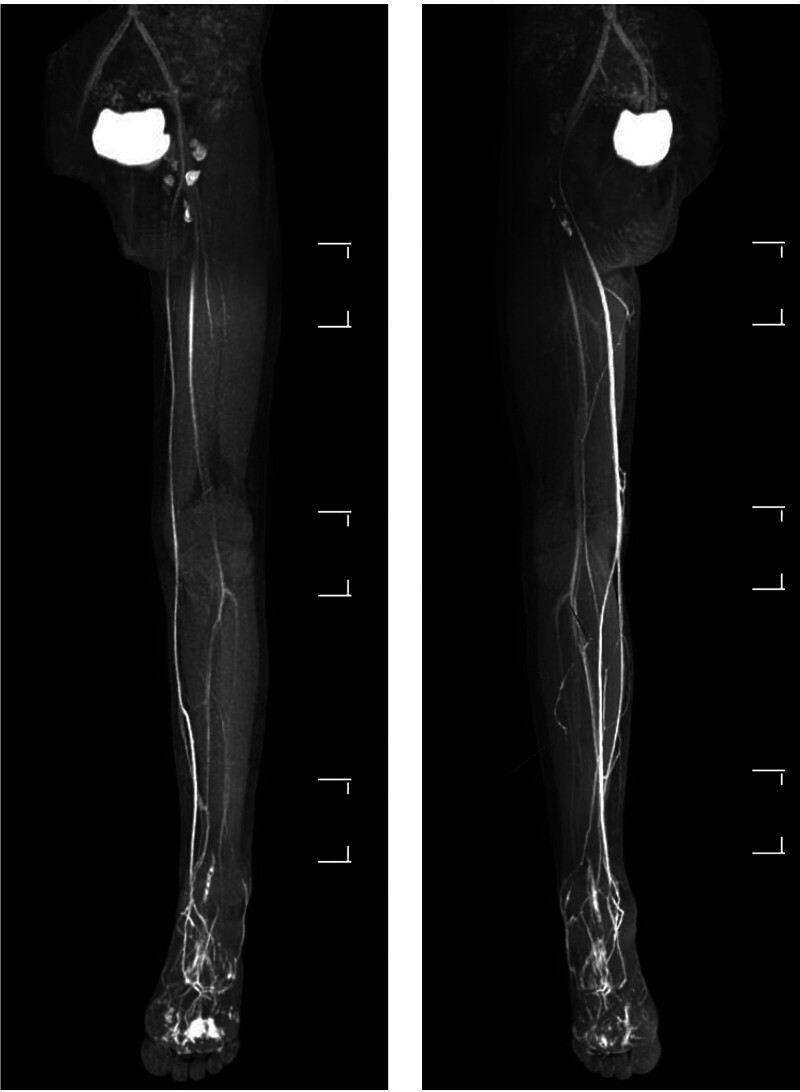
Coronal post-contrast MRI images of the lower extremities. The left panel shows the left leg, and the right panel shows the right leg. There is dermal backflow visible in the distal aspect of both legs, most notably along the dorsal surface of the right foot (red arrow), which appears faint and discontinuous—an atypical presentation likely due to early-stage dermal reflux. Gadolinium uptake is noted in the inguinal lymph nodes bilaterally, and mild soft tissue swelling is more pronounced in the right leg.

**Fig. 5:**
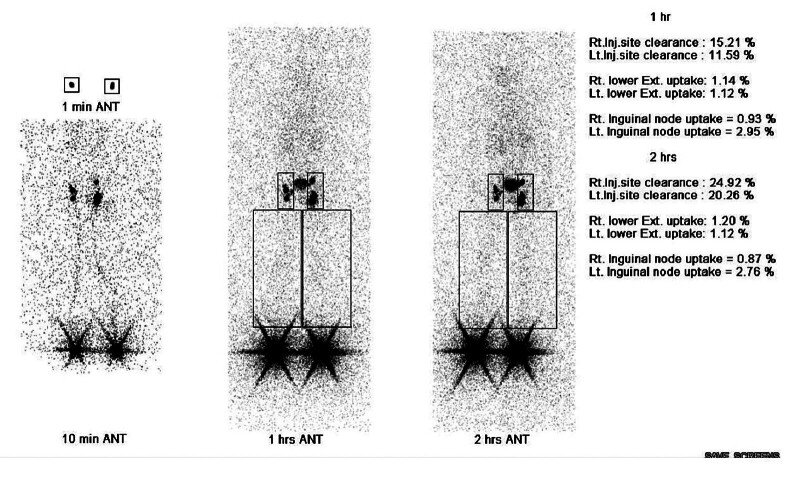
Lymphoscintigraphy at 10 minutes, 1 hour, and 2 hours post-injection (left to right). Early dermal backflow is seen bilaterally at 10 minutes. At 1 and 2 hours, persistent dermal backflow is more prominent in the right leg, with delayed tracer clearance and reduced right inguinal node uptake (0.93% at 1 hour; 0.87% at 2 hours) compared to the left (2.93% at 1 hour; 2.76% at 2 hours), indicating right lower extremity lymphedema.

**Fig. 6:**
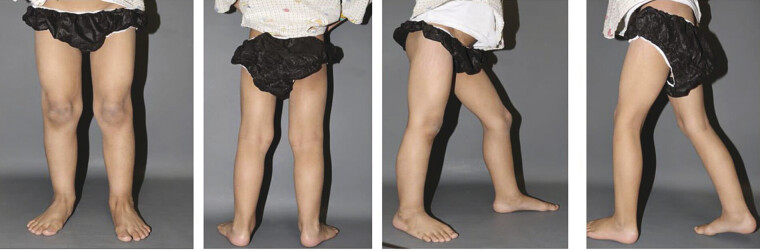
Preoperative images showing visible swelling and asymmetry in both lower extremities, with more pronounced lymphedema in the right leg.

### Surgical Intervention

The patient underwent bilateral lower limb distal LVA and bilateral groin LNVA, aimed at decompressing elevated intralymphatic pressure. By creating a shunt, the procedure effectively reduced abnormal lymphatic pressure, thereby minimizing lymphatic leakage into the intestines. This approach targeted the underlying cause of PLE, as increased subserosal lymphatic pressure was contributing to protein loss. Intraoperatively, the lymphatic vessels appeared fibrous. Moderate fibrosis was observed in the subcutaneous tissue, along with thickening of the fat lobular septa. Side-to-end lymphovenous anastomoses and side-to-end lymph node–vein anastomoses were performed using 11–0 and 10–0 nylon sutures, respectively. Immediate post-shunting patency was confirmed with ICG filter activation under the operating microscope, showing spontaneous lymphatic flow into the venous system. The surgery was performed without complications.

### Postoperative Course and Follow-Up

**Fig. 7:**
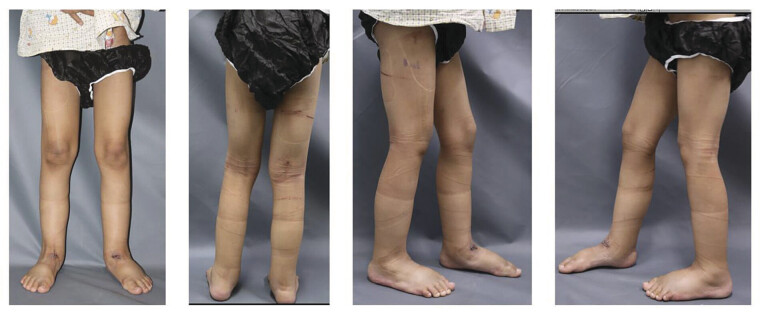
Postoperative images showing a significant reduction in swelling and improved symmetry in both lower extremities.

One week postoperatively, the patient demonstrated marked clinical improvement. Serum albumin levels increased to 2.8 g/dL, and peripheral edema and lower limb swelling significantly decreased. Gastrointestinal symptoms, including diarrhea and abdominal distention, also improved, and his nutritional status began to stabilize. Serial assessments of total protein and stool alpha-1 antitrypsin levels were performed. Total protein increased from 5.1 to 5.5 g/dL, and stool alpha-1 antitrypsin levels normalized, dropping from 136.7 to <26.00 within 1 month. Similarly, alpha-1 antitrypsin clearance, initially elevated at 138.7 mg/dL (normal: <27 mg/dL), normalized to <15.96 mg/dL within the same period. In the following weeks, albumin levels continued to rise, eventually reaching 3.2 g/dL, indicating sustained reduction in protein loss. Follow-up MR lymphangiography demonstrated resolution of abdominal lymphatic leakage. The patient experienced weight gain, improvements in muscle mass, and a reduced need for hospitalizations and nutritional interventions. The preoperative dietary protocol (high-protein, low-fat) was maintained throughout the postoperative period ( Fig. 7 ). The patient was maintained on a high-protein, low-fat diet supplemented with medium-chain triglycerides both before and after surgery to reduce intestinal lymphatic load. During the 3-year follow-up, the patient required albumin infusions only during episodes of upper respiratory infection, when protein and albumin levels dropped. After recovery, protein and albumin levels returned to baseline, and no further infusions were needed.

## Discussion

**Fig. 8:**
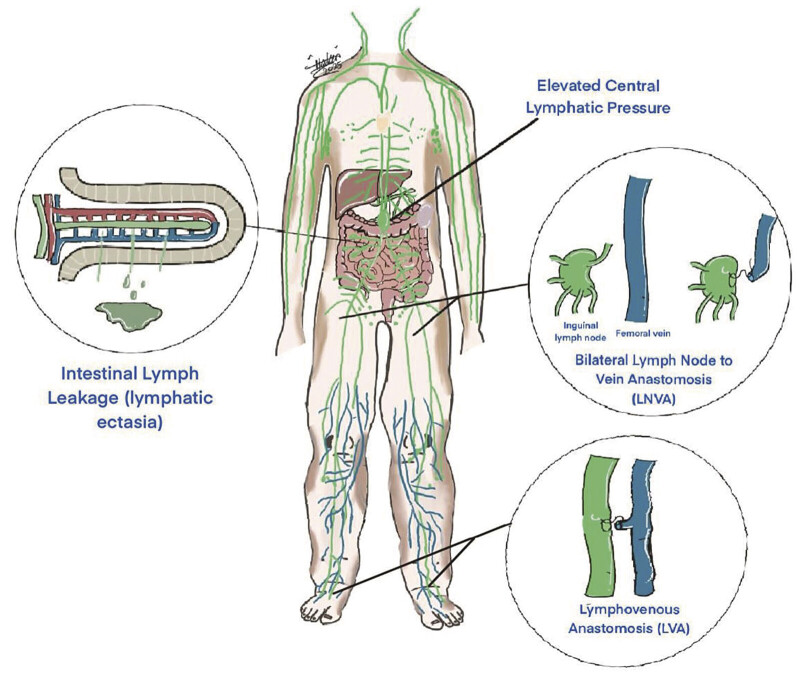
Illustration of lymphatic dysfunction and surgical treatment in PLE. Intestinal lymphatic leakage results from elevated central lymphatic pressure. Lymphatic decompression was achieved through bilateral LVA in the lower limbs and LNVA in the groin, thereby redirecting lymphatic fluid into the venous system. PLE, protein-losing enteropathy.

This case report demonstrates the successful use of lymphovenous bypass, and lymph node HSD1 mutations are known to be associated with both peripheral and central lymphatic abnormalities, leading to lymphatic dysfunction and contributing to conditions such as PLE. 10 This case report details the successful application of these novel surgical interventions for PLE in a pediatric patient with a THSD1 mutation. The 3-year follow-up indicates that the patient's albumin levels dropped to preoperative levels during a severe upper respiratory infection and have remained suboptimal despite the resolution of the infection. This finding underscores the possibility that systemic inflammatory episodes may transiently overwhelm the lymphatic system, leading to a regression of the condition. Despite the recurrence of hypoalbuminemia, the normalization of albumin levels immediately postoperatively without significant dietary or pharmacological changes reinforces the potential efficacy of LVA and LNVA in addressing PLE-associated lymphatic dysfunction. The likely mechanism underlying the improvement in PLE is the reduction of elevated central lymphatic pressure through peripheral decompression. By diverting lymphatic flow via LVA and LNVA, the surgery may have alleviated pressure in the thoracic duct and intestinal lymphatics, thereby reducing protein loss into the gastrointestinal tract ( Fig. 8 ). No liposuction was performed. Improvement is attributed to LVA and LNVA, which likely reduced central lymphatic pressure contributing to PLE. Although medical and dietary management were continued postoperatively, the immediate and sustained improvements in serum albumin and protein loss occurred without any modification to these measures, suggesting that the surgical lymphatic decompression was the primary driver of clinical improvement. Postoperative care protocols, as highlighted by Chan et al, emphasize the importance of avoiding immediate compression and promoting limb elevation to support lymphatic flow across newly created anastomoses, reducing the risk of thrombosis and improving outcomes. 10 This case highlights the importance of considering growth and systemic inflammation as potential triggers for recurrence, and future studies should explore tailored interventions for pediatric patients undergoing lymphatic surgery. This approach aligns with the findings of Kim et al, who demonstrated that lymphatic bypass procedures can be a minimally invasive, physiologically effective strategy to address evolving lymphatic pressure dynamics in pediatric patients. 11

THSD1 mutations are known to be associated with both peripheral and central lymphatic abnormalities, leading to lymphatic dysfunction and contributing to conditions such as PLE. 10 In this case, the patient's lymphatic abnormalities, driven by the THSD1 mutation, were central to the pathogenesis of his PLE. Given the refractory nature of the condition to conventional medical management, including nutritional support and albumin infusions, the decision to pursue lymphatic surgery was based on the clear association between the mutation and the lymphatic dysfunction. This highlights the potential of lymphatic surgery as a valuable therapeutic option for managing PLE in patients with congenital or genetic lymphatic abnormalities, particularly when medical therapies prove insufficient.

Lymphatic surgery, particularly LVA and LNVA, has gained recognition as an effective treatment for lymphedema, 7 12 but its use in managing PLE is novel and scarcely reported in the literature. In addition to our case, two recent studies demonstrated the successful application of these peripheral techniques in managing central lymphatic abnormalities. In one case, a child with chylothorax due to traumatic thoracic duct injury experienced a significant reduction in thoracic duct pressure and clinical improvement following LVA and LNVA. 13 Another study reported on a series of patients with Milroy disease complicated by congenital chylothorax and lymphedema, where peripheral decompression via LVA and LNVA led to improvement in both chylothorax and lower extremity lymphedema. 14 In both cases, elevated central lymphatic pressure—whether from traumatic injury or congenital failure of natural drainage pathways—was alleviated by peripheral decompression, resulting in symptom improvement. In our case, the patient's history of NICU admission, respiratory support, limb lymphedema, and narrowing of the distal thoracic duct on MRL all suggested increased central lymphatic pressure. The peripheral approach using LVA and LNVA most likely decompresses the lymphatic system pressure, decreasing the leakage, which leads to a reduction of protein loss and clinical improvement.

The role of lymphatic dysfunction in the pathogenesis of PLE has been investigated in previous studies, particularly in cases of primary intestinal lymphangiectasia, where abnormal lymphatic vessels contribute to protein loss through the gastrointestinal tract. 1 2 While conservative treatments like albumin infusions and nutritional support are commonly used, they often fail to address the underlying lymphatic abnormalities. 3 In our case, we successfully employed lymphovenous bypass and LNVA to treat a 7-year-old male with chronic PLE associated with a THSD1 mutation. Despite conventional management, the patient had persistent hypoalbuminemia and severe edema. Postoperatively, his albumin increased to 2.8 g/dL, with significant reductions in peripheral edema and gastrointestinal symptoms. Total protein improved from 5.1 to 5.5 g/dL, and stool alpha-1 antitrypsin levels and clearance normalized within 1 month. Over the subsequent weeks, albumin levels continued to rise, reaching 3.2 g/dL, with sustained improvements in protein loss, weight gain, and muscle mass with an important emphasis on maintaining the preoperative high-protein, low-fat dietary protocol throughout the management course to help further alleviate the lymphatic load.

The successful application of lymphatic surgery in our case aligns with previous reports exploring surgical approaches for managing PLE with lymphatic involvement. Jamal described a case of PLE associated with peripheral lymphedema, successfully treated with a lymph node–venous shunt and debulking surgery for leg and scrotal edema. 8 Similarly, Miyakawa et al reported a case of intestinal lymphangiectasia with refractory gastrointestinal bleeding, where intra-abdominal lymphaticovenous anastomosis and venous ligation led to dramatic improvement and sustained resolution of both PLE and gastrointestinal bleeding. 9 These cases, along with ours, suggest that various lymphatic surgical techniques, including lymphaticovenous shunts and anastomoses, can effectively manage PLE, particularly when conservative therapies fail to address the underlying lymphatic dysfunction.

The strength of this case lies in the detailed evaluation and tailored application of lymphatic surgery, specifically lymphovenous bypass and LNVA, to treat PLE related to a THSD1 mutation—a condition rarely managed surgically. If recovery does not occur, additional lymphatic decompression, such as expanded LVA or LNVA procedures, may be necessary to restore lymphatic equilibrium. This aligns with findings in pediatric patients with complex lymphatic abnormalities, where iterative procedures showed sustained efficacy over time. 2 3 Long-term, multicenter studies are needed to establish robust guidelines for managing PLE with lymphatic surgery in genetically predisposed populations. The 3-year successful outcome demonstrates the potential of this approach to more effectively address underlying lymphatic dysfunction in comparison to conventional therapies. A key strength is the adherence to CARE guidelines, ensuring thorough and standardized reporting of clinical details, interventions, and outcomes. 15 Perhaps even a longer follow-up may be warranted to see the durability of this approach, especially through adolescence. Additionally, as this is a single case, broader studies are necessary to confirm the generalizability of these results across diverse patient populations. Nevertheless, the rarity of this condition justifies the single case report, as no previous reports have detailed such a surgical approach for PLE related to THSD1 mutation.

### Conclusion

This case suggests that LVA and LNVA can improve PLE associated with lymphatic dysfunction in a pediatric patient with a THSD1 mutation, though recurrence during systemic inflammation highlights the need for further study.
